# Leaving No One Behind: Interventions and Outcomes of the COVID-19 Vaccine Maximising Uptake Programme

**DOI:** 10.3390/vaccines10060840

**Published:** 2022-05-25

**Authors:** Ilhem Berrou, Kathryn Hamilton, Clare Cook, Clare Armour, Sian Hughes, Jude Hancock, Sally Quigg, Huda Hajinur, Seema Srivastava, Charlie Kenward, Amjid Ali, Laura Hobbs, Elena Milani, Nicola Walsh

**Affiliations:** 1 Glenside Campus, School of Health and Social Wellbeing, University of the West of England, Bristol BS16 1DD, UK; nicola.walsh@uwe.ac.uk; 2 South West England Public Health Training Scheme, First Floor, Park House, 1200 Bristol Parkway North, Newbrick Road, Bristol BS34 8YU, UK; kathryn.hamilton2@nhs.net (K.H.); clare.cook@nbt.nhs.uk (C.C.); clare.armour@nbt.nhs.uk (C.A.); seema.srivastava@nbt.nhs.uk (S.S.); 3 National Health Service (NHS) Bristol, North Somerset and South Gloucestershire CCG, 360 Bristol, Marlborough Street, Bristol BS1 3NX, UK; sian.hughes21@nhs.net (S.H.); jude.hancock@nhs.net (J.H.); sally.quigg1@nhs.net (S.Q.); charlie.kenward@nhs.net (C.K.); 4 Caafi Health, Unit 18, The Coach House, 2 Upper York Street, St Paul’s, Bristol BS2 8QN, UK; huda79haji@gmail.com; 5 NHS Blood and Transplant 500, North Bristol Park, Filton, Bristol BS34 7QH, UK; amjidali@talktalk.net; 6 Department of Applied Sciences, Frenchay Campus, University of the West of England, Bristol BS16 1QY, UK; laura5.hobbs@uwe.ac.uk (L.H.); elena.milani@uwe.ac.uk (E.M.)

**Keywords:** COVID-19, vaccine hesitancy, minority ethnic groups, refugees, asylum seekers, Gypsy Roma Travelers communities, boat people, outreach vaccination, community-based interventions, community co-design

## Abstract

The devastating impact of COVID-19 on individuals and communities has accelerated the development of vaccines and the deployment of ambitious vaccination programmes to reduce the risks of infection, infection transmission and symptom severity. However, many people delay or refuse to get vaccinated against COVID-19, for many complex reasons. Vaccination programmes that are tailored to address individual and communities’ COVID-19 concerns can improve vaccine uptake rates and help achieve the required herd-immunity threshold. The Maximising Uptake Programme has led to the vaccination of 7979 people from February–August 2021 in the South West of England, UK, who are at high risk of severe illness from COVID-19 and/or may not access the COVID-19 vaccines through mass vaccination centres and general practices. These include: people experiencing homelessness; non-English-speaking people; people from minority ethnic groups; refugees and asylum seekers; Gypsy, Roma, Travelers and boat people; and those who are less able to access vaccination centres, such as people with learning difficulties, serious mental illness, drug and alcohol dependence, people with physical and sensory impairment, and people with dementia. Outreach work coupled with a targeted communication and engagement campaign, co-designed with community leaders and influencers, have led to significant engagement and COVID-19 vaccine uptake among the target populations.

## 1. Introduction

In December 2019, the first reports of a severe respiratory infection emerged from Wuhan, China. Samples from the affected patients revealed a novel virus, SARS-CoV-2, as a cause of the Coronavirus Disease 2019 (COVID-19). Soon after, similar presentations were being reported globally. In the UK, the first cases were identified in late January 2020. In March of that year, the World Health Organisation (WHO) declared COVID-19 as a global pandemic [1]. The UK COVID-19 death toll is among the highest in the world [2], with more than 162,000 deaths recorded from the start of the pandemic through to March 2022 [3]. People living in deprived areas and those from ethnic minority groups had higher diagnosis rates and death rates, which confirmed the impact of COVID-19 on replicating and widening the existing health inequalities [4]. The disproportionate impact on disadvantaged and ethnic communities has also been reported in other countries such as France [5], Italy [6], Spain [7] and the USA [8].

The devastating impact of the virus has accelerated the development of COVID-19 vaccines in order to reduce the risks of infection, infection transmission and symptom severity [9,10,11]. The UK national COVID-19 immunisation programme was launched in December 2020, aiming to vaccinate every adult, and more recently every age-eligible child, in the country. Furthermore, individuals from certain ethnic minority groups and those experiencing socioeconomic deprivation were identified to be at high risk of COVID-19-related morbidity and mortality and were therefore recommended to be prioritised in vaccination programmes, and targeted by effective health promotion campaigns. [12]. However, vaccine hesitancy, defined as delaying or refusing the vaccine despite the availability of vaccine services [13], may hinder the successful implementation of the vaccination programme and achieving the required herd-immunity threshold [14]. 

Low confidence in COVID-19 vaccines is prevalent among many Black, Asian and minority ethnic groups, those who live in deprived areas, and those with no education qualifications [15,16]. Throughout the course of the pandemic, vaccine hesitancy improved slightly, with many people engaging with national vaccination programmes. However, low confidence in COVID-19 vaccines still exists among ethnic minority groups and those experiencing socio-economic deprivation [17].

The reasons behind COVID-19 vaccine hesitancy are complex but can be articulated using the 5C framework: Confidence (lack of trust in the safety and effectiveness of the vaccine), Complacency (not perceiving diseases as high risk and vaccination as necessary), Constraints (having practical barriers to getting vaccinated), Calculation (engagement in extensive information searching and deliberation), and Collective responsibility (willingness to protect others through own vaccination) [18]. Analysis of data from “Understanding Society”, a large UK household longitudinal study, show that Black or Black British participants were more likely to report that they do not trust the vaccines, while Pakistani or Bangladeshi participants were more worried about the vaccines’ side-effects. Vaccine hesitancy was also found to be associated with lower education attainment [15]. Many barriers to COVID-19 vaccination have been reported. These include the rapid development and approval of the COVID-19 vaccines, lack of trust in officials and in the vaccine itself, safety concerns, perceptions of COVID-19 risks, (mis)information and conflicting/changing health messages, lack of diversity in studies assessing the vaccine and in public messages, and transparency of the vaccination programme [19,20,21,22]. The same issues relating to trust, misinformation, and lack of diversity have also been implicated in hesitancy to access the influenza vaccine and other vaccines [23,24,25].

Studies seeking to understand COVID-19 vaccines’ uptake trends and reasons for vaccine hesitancy have brought to light the effect of health inequalities on vaccine hesitancy—a problem that has existed since long before the pandemic [26]. While it is important to continue exploring the depths of this issue, it is paramount that we also explore how public health interventions are implemented, on the ground, to address those inequalities and improve vaccine uptake. A recent study by Thorneloe et al. [27] highlighted, through workshops with NHS staff, the importance of information on the benefits of vaccines, addressing concerns about safety and risks, maximising the visibility and accessibility of relevant vaccine information¸ and ensuring support in the workplace to receive and recover from the vaccine side effects. Bateman et al. [28] reported that the use of an SMS (short message service) containing a vaccine information video increased rheumatoid arthritis patients’ information on COVID-19 vaccines, which affected their confidence and intention to have them. Salali and Uysal [29] assessed the impact of a range of incentives on people’s intention to get a COVID-19 vaccine in the UK, USA and Turkey. They highlighted that vaccinating an expert scientist, friends and family members and knowing someone dying from the disease are strongly associated with increasing peoples’ intention to get the vaccine. 

Apart from this small number of studies, we know very little about effective practical approaches to improve the uptake of COVID-19 vaccines in hesitant groups. Lessons learnt from vaccination programmes targeting areas, people and groups with low rates of COVID-19 vaccine uptake provide valuable insights into how vaccine opinions and attitudes could be changed. In the present study, we explore the interventions and outcomes of the Maximising Uptake Programme, designed and coordinated by the Maximising Uptake Group, and commissioned by the Healthier Together partnership for Bristol, North Somerset and South Gloucestershire (BNSSG), UK, to increase COVID-19 vaccine uptake in groups with low vaccine uptake rates in the region. 

## 2. Materials and Methods

### 2.1. BNSSG CCG COVID-19 Maximising Uptake Programme

Located in the Southwest of England, BNSSG is home to a mix of urban and rural populations of almost one million. Around 16% of the Bristol population are minority ethnic people, compared to 5% in South Gloucestershire and 2.7% in North Somerset [30]. Within BNSSG, there are communities living in significant deprivation, mainly focused in Bristol, but found in pockets throughout BNSSG. In two of the most deprived areas in Bristol, Lawrence Hill and St Paul’s, 60 and 80% of the population, respectively, belong to a minority ethnic group [31]. It is estimated that around 1 in 10 people in BNSSG live in a deprived area [32]. Furthermore, people in the most deprived areas live on average 6–15 years less than those who live in the least deprived areas [30]. Notably, ‘deprivation’ is defined by the IMD (index of multiple deprivation) of the small area (LSOA) in which an individual lives. The home postcode of individuals is used to look at the deprivation code (1–10) of the area they live in [33]. 

Initial insights from the local COVID-19 vaccination networks and community teams at the start of the COVID vaccination programme confirmed the low levels of uptake among ethnic minority groups and under-served communities. To address this issue, a dedicated group within the regional COVID-19 Vaccination Programme, called the Maximising Uptake Group (MUG), was set up in December 2020 to design and coordinate the Maximising Uptake Programme to increase vaccine uptake. The priority groups for the MUG were:

Group 1: People experiencing homelessness.

Group 2: Non-English-speaking people, people from minority ethnic groups, refugees and asylum seekers—particularly concentrated in the Bristol Inner City area. 

Group 3: Gypsy, Roma, Travelers (GRT) and boatpeople. Communities that find it hard to reach vaccination centres.

Group 4: Those who are less able to access vaccination centres such as people with Learning Difficulties (LD), Serious Mental Illness (SMI), Drug and Alcohol (DA) dependence, people with physical and sensory impairment and people with dementia.

Hospital patients were initially included as a target group within the MUG programme but were later moved under a different COVID-19 vaccination programme. The development of the programme is described in [34] and has continued to evolve depending on the nationally-driven programme direction and the real-time local uptake data. 

### 2.2. Study Design

This was a retrospective descriptive cohort study. We used:

- COVID-19 vaccine uptake data from BNSSG Clinical Commissioning Group (CCG) and vaccine update data among national priority subgroups from the national vaccination uptake data system from December 2020 to May 2021.
- Data on numbers, geographic locations and vaccinations delivered during the outreach and engagement activities supplied by the MUG leads, BNSSG CCG, and Sirona Care and Health (a local community healthcare provider).
- Routinely collected quantitative and qualitative data, supplied by the BNSSG CCG Insights and Engagement team, between December 2020 and May 2021, including before and after outreach/engagement brief surveys, online public engagement surveys and vaccination follow up interviews with people from vaccine priority cohorts. These engagement surveys and interviews were carried out before and after outreach clinics to explore people’s satisfaction with the vaccination clinics and identify areas for development to inform the organisation and delivery of future clinics.

From May–June 2021, semi-structured interviews were undertaken online due to the need for social distancing during the data collection timeframe [35], with 35 MUG programme leaders, programme facilitators, and local communities’ representatives and leaders (the interview guide is presented in Supplementary File S1). The interviews lasted for 25 to 60 min. Most interviews were one to one, but a minority involved two or three programme collaborators from within the same sub-section of the programme. Some programme leaders spoke with others working in their area before their interview to gather views and feedback. Interviews were transcribed and data were thematically analysed to draw out the lessons learnt from the programme implementation, remaining challenges and recommendations for future interventions.

### 2.3. Statistical Analysis

Descriptive statistics including percentages and frequencies were used to present the number of outreach clinics and COVID-19 vaccinations delivered between December 2020 and May 2021. The percentage uptake of the COVID-19 vaccine was calculated using the number of people who have received a vaccine, divided by the total eligible population within BNSSG. The percentage uptakes are shown against the total population average (so 0 is the average and above or below this is the % away from the average). The numerator and the denominator values were obtained from the BNSSG system-wide dataset. The vaccine data in the system-wide dataset are based on GP clinical systems which are updated from the National Immunisation Management System (NIMS). 

### 2.4. Ethical Approval

The BNSSG Mass Vaccination Clinical Delivery Group (CDG) and BNSSG CCG NHS Clinical Effectiveness and Research group approved this study. The study adhered to BNSSG CCG’s research governance policies, regulations and standards [32].

## 3. Results

The MUG programme included two key interventions: engagement and communication with people and communities to change opinions and debunk misinformation and myths about the COVID-19 vaccine; and focused outreach activities (pop up clinics and other outreach work) to get the vaccine where people can easily access it. Vaccine uptake data were closely monitored to identify groups/areas falling behind in terms of COVID-19 vaccine uptake rates and to target interventions to close emerging gaps. 

### 3.1. Engagement and Communication with People and Communities

These activities were carried out before or during outreach activities. The MUG approach to engagement and communication with the target groups was tailored to meet the needs of the specific groups. Engagement and communication with Group 1 and Group 3 populations were verbal and undertaken by NHS healthcare professionals, trusted support workers and volunteers known to the population in these groups. In contrast, communication and engagement with Group 2, the biggest target group for the programme, involved written materials and social media outputs, in different languages, delivered by local community influencers, community and faith leaders, friends and family members, and minority ethnic healthcare professionals. These were disseminated through local ethnic media channels, community social media sites and community venues. Further details on the engagement and communication strategy, particularly for Group 2, are shown in Supplementary File S2 Box S1.

### 3.2. Ourtreach Activities 

A total of 7979 vaccinations were given through 162 outreach activities, as shown in Table 1. A BNSSG CCG insights survey involving 500 people attending outreach community clinics showed that 15.5% had already been offered a vaccination appointment, and that 39% strongly preferred to attend the local clinic rather than an NHS facility. Around 67% of survey respondents attending one community clinic did not want to attend a GP practice or mass vaccination centre to get vaccinated [36].

#### 3.2.1. Group 1: Homeless People

Prior to carrying out outreach activities with this population, BNSSG CCG conducted semi-structured interviews with 22 homeless people from October–November 2020 in Bristol and Weston-Super-Mare in order to explore their perceptions and attitudes towards COVID-19 vaccinations and how best to meet their needs. A summary of findings relevant to the design of outreach activities with this group is shown in Supplementary File S2 Box S2. Taking those insights into account, a number of outreach activities were carried out as follows:

- Onsite clinics at hotels, hostels and other accommodations
- Engagement with homeless people, on the street, though trusted individuals and homeless support workers

For those in dispersed housing, registration with a GP was encouraged to ensure increased access to vaccination through their GP. There was also emphasis on raising awareness of the importance of GP registration amongst staff in homeless housing options. 

**Figure 1:**
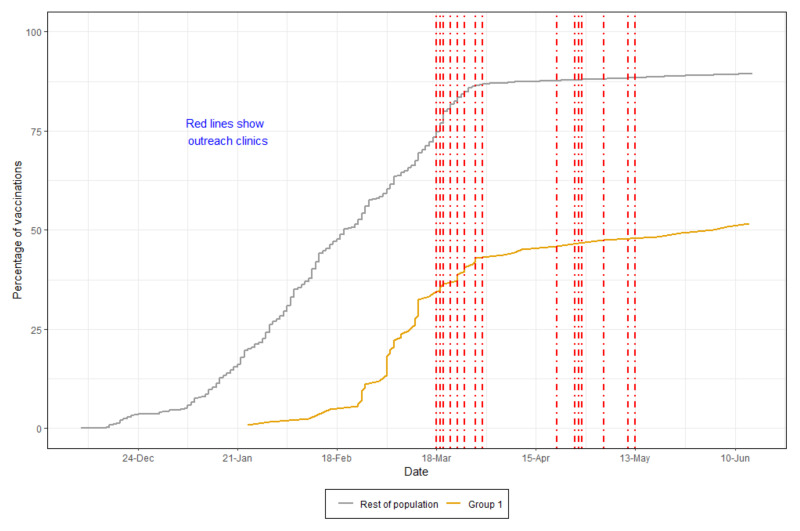
Vaccination rates (first vaccines) for homeless people compared to the BNSSG population. The red dotted lines represent some of the outreach activities.

There were 199 people registered as homeless on the BNSSG local dataset. However, this is likely to be an under-estimation of the numbers of homeless people in the area. Of those, 47.3% of people received their vaccine (to 17 May 2021), compared to 84.4% of the whole population in the area, as shown in Figure 1. As outreach workers were going around vaccinating homeless people on the street, shelters, and emergency accommodation, they came across more homeless people, most of whom were not registered on any database. Due to this, the number of vaccines given exceeds the number of registered homeless people and includes first and second vaccine doses.

The BNSSG insights team carried out a number of structured interviews and surveys with homeless outreach clinics’ workers in May and June 2021 to evaluate the outreach and engagement activities undertaken with this group and to highlight issues to consider in future interventions targeting this group. Themes emerging from this evaluation activity are summarised in Supplementary File S2. Taking the vaccine to where homeless people congregate and/or sleep, delivering the vaccine through homeless support workers and organisations that usually support homeless people, and opportunistic vaccinations even if outside the priority criteria at the time increased homeless peoples’ engagement with the programme. However, many challenges persist in relation to increasing the uptake of the COVID-19 vaccine in this group. Data on homeless people are scarce in healthcare and local authority records. Many homeless people may not trust authorities and may not have or be able to access services to support them. Future national vaccination programmes should set clear criteria for eligibility based on homelessness. 

#### 3.2.2. Group 2: Non-English-Speaking People, People from Minority Ethnic Groups, Refugees, and Asylum Seekers

Insights from pilot pop-up influenza clinics delivered in a mosque and community centre and the feedback from community engagement activities suggested that using community spaces to deliver vaccinations encourages people to get vaccinated. Furthermore, people in this group are more likely to attend vaccination clinics if bookings and appointments are arranged by local community groups instead of health organisations. Based on this, a number of successful outreach clinics were delivered across the BNSSG area in hotels, mosques, community centres, churches, supermarkets, shops, parks and other facilities. These required close collaboration with community leaders, faith leaders, and local influential business owners.

**Figure 2:**
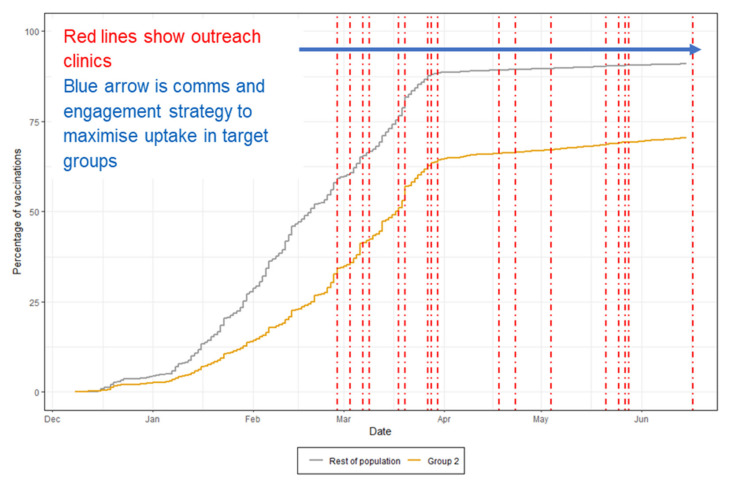
Percentage of population vaccinated (first doses) for Group 2 vs. the rest of the BNSSG population. The red dotted lines represent some of the outreach activities.

There are 126,710 people in Group 2. As of May 2021, 61.3% of eligible people who are non-English speaking or in minority ethnic groups were vaccinated (including through mass vaccination centres and GP practices), compared to 84.4% of the whole BNSSG population. This is shown in Figure 2. Figure S1 in Supplementary File S3 further illustrates the increase uptake of vaccination, coinciding with the delivery of the programme interventions. 

The MUG delivered regular focus groups and informal conversations within the community led by trusted healthcare professionals in order to motivate community members to get vaccinated. Furthermore, the local community influencers and leaders (called community champions) were responsible for managing a booking system that enabled many COVID vaccine “pop-up” clinics in community centres, mosques and gurdwaras. Some clinics had the capacity to vaccinate up to 250 people per session. The community champions also co-designed information in multiple languages and in different media (text message, leaflets, videos, and posters) to be delivered throughout the campaign. 

Link workers who spoke different languages were key to supporting people arriving to the clinics. Furthermore, in many of those clinics, separate rooms were set up for women to be vaccinated in a private area, respecting the communities’ religious beliefs and practices. Those who attended the clinics were encouraged to “spread the word” amongst their friends and family using their personal social media channels and conversations. The BNSSG CCG team encouraged local TV coverage from popular ethnic minority channels to increase the dissemination of the campaign messages. Post-vaccination feedback surveys in multiple languages were disseminated to optimise and improve service delivery in vaccination clinics. Notably, many of the clinics’ attendees reported that they would not have otherwise attended a vaccination clinic, especially at a mass vaccination centre, given that the centres did not have interpreters, and many people, especially those who lived in Bristol Inner City, did not own cars, were not able to drive and could not arrange childcare. 

**Figure 3:**
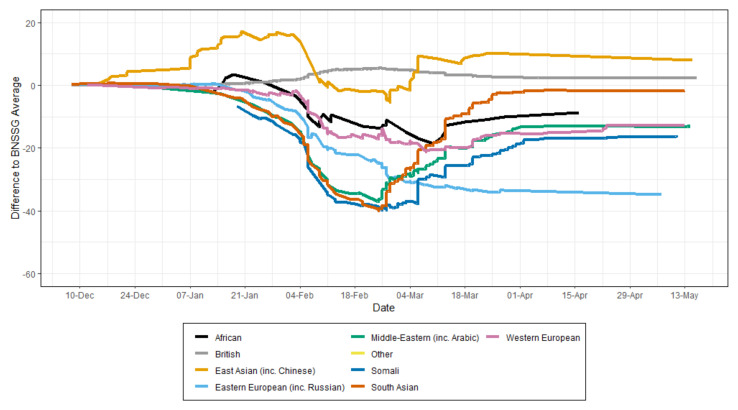
Percentage of vaccination uptake (first dose) by the first language group in the BNSSG area. JCVI (Joint Committee on Vaccination and Immunisation) priority groups for COVID-19 vaccination [37].

Members of Eastern European communities had the lowest levels of COVID-19 vaccine uptake in the area as shown in Figure 3. Community leaders were not easy to identify, and engagement with community events and activities was not substantial. Furthermore, local insights revealed widespread anti-vax messaging among the communities. Engagement insights further suggest that members of these communities had high levels of mistrust in government messages and efforts. The reasons for the low levels of engagement among these communities warrant further investigation.

Asylum seekers and refugees were vaccinated in clinics that were coordinated by the existing primary care service for these groups. There was a significant contribution from the voluntary sector in supporting these clinics. Undocumented migrants were also vaccinated at these clinics. During the vaccination programme, work was undertaken to streamline the process for GP registration for asylum seekers and refugees, as this was a barrier to follow up and addressing other health issues. People in these groups (particularly undocumented migrants) may not be visible to healthcare or other systems, and this represented challenges for registering vaccination status and following the vaccine schedule. 

Insights from semi-structured interviews with MUG programme leaders and facilitators, along with and post-clinics’ surveys and structured interviews with people from Group 2 who attended outreach clinics are summarised in Supplementary File S2. Recognising and harnessing the potential of community leaders and community spaces are key to increasing engagement with vaccination efforts. Furthermore, communicating in the right language, through the right channels, is important to reach people and increase their engagement with local public health efforts. Supplementary File S2 includes examples of BNSSG CCG communications with this group. 

#### 3.2.3. Group 3: Gypsy, Roma, Travelers (GRT) and Boatpeople

In addition to the Gypsy Roma Traveler (GRT) population, Group 3 also includes those living a distance from the vaccine centre, those living in areas of high deprivation, and rural communities. GRT and boat people are a small minority of Group 3 which included wider groups. The number of GRT and boat people in BNSSG area is unknown. GRT and boat people are less likely to be registered with health systems or local authorities for many reasons, including geographical mobility. Local data identified 226,148 people in Group 3. After reviewing initial uptake data, the focus of the MUG programme was on those living in areas of high deprivation (data shown in Figure S2 in Supplementary File S3) and Gypsy Roma Traveler populations. For GRT and boat people, a total of 132 COVID-19 vaccinations were delivered through 13 outreach activities. 

Insights from semi-structured interviews and questionnaires with programme workers and GRT community leaders carried out in April–May 2021 show that, overall, there was a good level of engagement with the outreach clinics. A summary of the factors identified as strong facilitators of engagement is shown in Supplementary File S2. 

Notably, some traveler communities, e.g., New Age Travelers, may not have community leaders, and hence, may not engage well if the campaign communication approach is not versatile and adaptive. Furthermore, some communities exhibit strongly held vaccine concerns, especially around fertility among the Irish Travelers community, and would therefore require a dedicated communication strategy to address them.

#### 3.2.4. Group 4: Those Who Would Struggle to Access Vaccination Centres, Such as People with Learning Difficulties (LD), Serious Mental Illness (SMI), People with Physical and Sensory Impairment, People with Drug & Alcohol (DA) Dependence, and People with Dementia

In the BNSSG area there are 111,898 people in this group. The work for this priority group was coordinated by a lead GP for the GP collaborative board. Partners involved were Sirona Care and Health, Primary Care Networks (PCNs) and GP practices, Avon and Wiltshire Mental Health Partnership NHS Trust (AWP), Drug and Alcohol Services and others. The majority of people in this group were vaccinated by GP practices/PCNs and the large-scale vaccination sites. However, the MUG team supported the teams to identify patients who may not be able to attend those vaccination sites. This was done through:

- Developing medical records searching strategies to identify patients, e.g., with SMI using the definition by the Quality and Outcomes Framework (QOF) and also prioritising people with eating disorders due to their clinical vulnerability.
- Adapting processes within large scale vaccination centres, Sirona Care and Health and PCN sites to accommodate the needs of people from this group—e.g., learning disability needs for appointment time, quiet setting and limited waiting.
- Maintaining regular communication with primary care teams through meetings and electronic bulletins.

Outreach clinics were not as useful for this group. However, service delivery was flexible. For example, GP data was used to identify patients who are not able to attend a vaccination centre, e.g., housebound patients, and home visits were arranged to vaccinate them. The MUG team also worked with other care providers, e.g., mental health services, to deliver vaccination as part of usual care, and key workers for people with SMI. More than 89% of this population received their first vaccine dose (to 17 May 2021) compared to 84% of the whole population. The vaccine uptake in this group is overall high and has been throughout the programme. 

It is important to note that due to relying on GP healthcare records or community support services to identify and serve people in this group, it is possible that some people have been missed, especially those with mild learning disabilities, as these are less likely to be recorded and/or involve carers. In addition, people with complex needs, e.g., drug and alcohol dependence, homelessness, and mental illness, may not be visible on the records system. Therefore, observing overall uptake numbers will not identify uptake issues for smaller numbers of people with multiple barriers and vulnerability issues; those are likely to require intensive input/inclusion health work.

### 3.3. Programme Costs

The whole programme costs (including communication and engagement work) are estimated at £15 additional cost per dose delivered through the outreach activities. However, considering the prioritisation of high-risk individuals who are reluctant to get vaccinated and the potential prevention of high-cost hospital admissions, this programme is likely to be cost-effective to the NHS. In BNSSG, an average intensive care unit stay costs £1283 per bed day. 

Notably, the above costs were calculated prior to delivering the larger walk-in outreach activities, and do not include the benefits to those who engaged with the programme but got vaccinated through the mainstream system. Therefore, the costs are likely to be less than the initial estimates. It is also important to acknowledge that cost-effectiveness is difficult to explore in this context, as an unknown number of people vaccinated in outreach settings would have been vaccinated at a later date in the mainstream programme. 

## 4. Discussion

This study presents findings on the interventions and outcomes of the Maximising Uptake Programme to increase COVID-19 vaccine uptake in population groups with low vaccine uptake rates in Southwest England, UK. The programme included a number of outreach activities consisting of pop-up clinics in parks, shops and community spaces, and targeted vaccinations in homeless shelters, emergency accommodations, and boats. Outreach work was coupled with a targeted communication and engagement campaign, co-designed with community leaders and influencers, and disseminated through community media and social media channels.

Research reporting on the implementation and outcomes of public health programmes to tackle COVID-19 vaccine hesitancy and increase vaccine uptake among individuals and communities is scarce. This research is among a few studies that contribute to the evidence base around co-designed, community-based interventions to tackle vaccine hesitancy. The Meharry Medical College Mobile Vaccination Programme [38] used mobile clinics and door-to-door outreach work to enhance COVID-19 vaccine uptake among disadvantaged and underserved communities in Middle Tennessee. The programme team highlights that mobile deployment of the vaccine and clinicians to deliver it are vital to meet the needs of people and communities that may not be able to access mass vaccination centres. Similar to our findings, the programme team recognises the importance of community engagement advocates to provide targeted, culturally competent information about the vaccines. Our findings are also consistent with [29] suggestions that effective vaccine messaging delivered by trusted experts and vaccinating family and friends can encourage hesitant individuals and communities to get the COVID-19 vaccine. Elements of this programme went further towards co-production with communities, and new ways of working were piloted in the extraordinary circumstances. For instance, some community leaders ran clinics including recruiting people for vaccination, with support from the programme. Handing over responsibility was a key element to building trust. This was enabled by a strong regional governance structure that supported innovation and safe multiagency working at pace. 

Tailored communication and outreach work have been key interventions in many successful COVID-19 vaccination programmes throughout the UK [39] and have also been advocated by the CDC. However, we need to recognise that this approach may not meet the needs of everyone. Strongly held negative beliefs about the side effects of the vaccine, especially beliefs of negative impact of the COVID-19 vaccines on fertility, still prevent some people from being vaccinated. This issue predates the COVID-19 pandemic. Bell et al. [40] highlighted the importance of understanding the context of people’s lives and how it shapes health decision-making when exploring Roma Romanian communities’ acceptance and uptake of vaccines against measles. Similar considerations were highlighted in the Smith and Newton study [41]. Taking the vaccine to the community and brokering access to the community through trusted midwives and nurses who have supported the community before and during the pandemic were key to improving vaccination rates in this group. 

Outreach work and communications with the Eastern European community in the area did not convince many members to get the COVID-19 vaccine. Our approach to co-design outreach interventions with community and faith leaders worked well with groups that have a clear faith and community leadership (Black, Asian and minority ethnic communities). However, faith leadership in the Eastern European community could not broker engagement from the members, and there were no overt leadership figures to collaborate with in this group. Although it is not entirely clear why our approach was less successful, Tankwanchi et al. [42] suggests various reasons for low acceptance of vaccines such as the influenza vaccines that could explain the low engagement with our programme. They suggest that Polish migrants continue to be influenced by Polish diaspora media and developments in Poland, particularly in relation to Russian-influenced anti-vaccine, anti-Western propaganda. This issue warrants further investigation to understand reasons for COVID-19 vaccine hesitancy in this community and develop effective interventions, especially as the UK is home to a large Eastern European community [42] that is vulnerable to the severe implications of COVID-19 infection. 

This study presents evidence of the impact of the Maximising Uptake Group’s programme on the target populations in the BNSSG area and provides ample practical recommendations to consider when designing and delivering vaccination interventions for underserved and disadvantaged communities. However, our findings have to be interpreted with caution. We used a retrospective descriptive cohort design to explore how the interventions were implemented and assess some of their intended outcomes. However, a cohort study design is a more appropriate approach to evaluate the effectiveness of the programme. The vaccination programme is unprecedented and in a unique time of national and global emergency. It is challenging to attribute changing vaccine uptake patterns to engagement interventions in this context; there are many national and local contextual factors to consider alongside the activities undertaken. Similarly, although numbers of vaccinations delivered in outreach settings have been described, and trends in uptake in priority groups have been mapped against outreach activities, most of the evidence only shows temporal association between overall vaccine uptake rates and outreach activities, and is therefore observational and limited. Only the direct impact of doses delivered through outreach work can be stated with certainty. There will be secondary impacts, as influence spreads within community networks. Quantifying these effects is the objective of a related local research project. The data presented here aggregates first and second doses delivered, and the priority groups include a broad range of ethnicities and other protected characteristics that are collapsed into a broader group, so it is hard to identify the impact on individual subgroups. The programme was developed and implemented in a public health emergency context, so evaluation of the programme was ongoing throughout the implementation process, and changes and “tweaks” were consistently incorporated into the programme.

## 5. Conclusions

The Maximising Uptake Programme has led to the vaccination of many people who were likely to be the most at risk of severe illness or death from COVID-19, and those who would not have been able to access mass vaccination centres and GP practices. The network of community partners established through of the MUG’s work has boosted the local organisations’ confidence and ambition to broaden the reach of vaccination efforts. The MUG’s programme has provided a strong foundation for future collaboration with community champions and providers of healthcare, local councils, and public health organisations, to support improvements in the health of underserved and ethnic minority populations. 

## Figures and Tables

**Table 1:** Number of outreach activities and COVID-19 vaccines given (including first and second doses) for each of the MUG target groups.

Target Group	Outreach Activities ^1^	Period	N Vaccines Given
Group 1: Homeless people	51	March–August 2021	504
Group 2: Non-English-speaking people, people from minority ethnic groups, refugees, and asylum seekers	93	February–August 2021	7241
Group 3: Gypsy, Roma, Travellers and boat people	13	March–August 2021	132
Group 4: People with LD, SMI, Physical and sensory impairments, D&A dependence, dementia	5	June–August 2021	102
Total	162		7979

^1^ Outreach activities include COVID-19 pop-up and mobile vaccine clinics. LD, learning difficulties; SMI, serious mental illness; DA, drug and alcohol dependence.

## Data Availability

The quantitative data presented in this study are available on request from the BNSSG analytics team (bnssg.analytics@nhs.net). Data access is subject to satisfaction of pre-set criteria and ethics review by the organisation.

## Supplementary Materials

The following supporting information can be downloaded at: https://www.mdpi.com/article/10.3390/vaccines10060840/s1. Supplementary File S1 includes an interview guide for programme workers and lists the members of the Maximising Uptake Group (MUG) contributors. Supplementary File S2 includes a number of text boxes summarizing the key themes and insights emerging from qualitative data. Supplementary File S3 includes additional data relating to vaccine uptake among Group 2 and 3 populations.
