# Supplementary material for: Leaving No One Behind: Interventions and Outcomes of the COVID-19 Vaccine Maximising Uptake Programme
Source: Vaccines (Basel). 2022 May 25;10(6):840. doi: 10.3390/vaccines10060840 (PMC9227842; doi:10.3390/vaccines10060840)
Supplement: Supplementary file 1 [file vaccines-10-00840-s001.zip › vaccines-1670294-supplementary.pdf]

Supplementary file S1

Interview guide for programme workers (adapted for community leaders)

Section 1: Learning from the programmes:

1. Please describe how the process of engagement was developed and identify any key people. You might consider –

a. How was early insight work or data used?

b. What was our relationship like with this community/group before the engagement work?

c. Were there key community leaders or partner organisations involved?

d. How did we learn as we went along?

2. Please describe what has worked well in engaging with, and increasing vaccination rates in one or more of the priority groups.

3. Please describe any barriers to successful engagement you have encountered – these might be practical, cultural, language, resources or otherwise. Have we managed to overcome these barriers?

4. Please describe how the maximising uptake programmes need to adapt moving forwards

5.How do the mass vaccination teams, the PCN teams and the maximising uptake teams work together to engage people and increase vaccination uptake?

6. Governance: is the governance process clear and straightforward? Does the relationship between programme management and those working directly with communities work well (and why)?

Section 2: About you:

7. What is your job title and role in the vaccination programme?

8. Do you work with, or have personal links to a particular priority group, that have been relevant to your work (please leave blank if you would rather not answer this)?

Maximising Uptake Group (MUG) contributors and evaluation team members

Dr Kathryn Hamilton	North Bristol NHS Trust/Public Health Training Scheme SW England
Dr Seema Srivastava	North Bristol NHS Trust
Dr Charlie Kenward	Bristol, North Somerset & South Gloucestershire CCG
Sian Hughes	Bristol, North Somerset & South Gloucestershire CCG
Clare Cook	BNSSG Programme Manager Mass Vaccination Outreach Programme
Emma Smith	BNSSG MUG Programme support
Cathy Holloway	Bristol, North Somerset & South Gloucestershire CCG
Sarah Hollier	Bristol, North Somerset & South Gloucestershire CCG
Thomas Manning	Bristol, North Somerset & South Gloucestershire CCG
Dr Jude Hancock	Bristol, North Somerset & South Gloucestershire CCG
Clare Armour	North Bristol NHS Trust
Carol Slater	Public Health, Bristol City Council
Finlay Mundy-Baird	University of Bristol Medical School
Dr Huzaifa Adamali	North Bristol NHS Trust
Prof. Nicola Walsh	University of the West of England
Dr Elena Milani	University of the West of England
Dr Laura Hobbs	University of the West of England
Dr Ilhem Berrou	University of the West of England
Huda Hajinur	Registered Nurse, Vaccine Coach and Community advocate
Amjid Ali	NHS Blood and Transplant and Kidney Care UK, Community advocate
Dr Shahnaz Chowdhury	Sirona Care & Health
Dr Kate Rush	Sirona Care & Health
Dr Caroline Crentsil	Sirona Care & Health
Anne Winter	Sirona Care & Health
Mary Lewis	Sirona Care & Health
Kate Cooke	Public Health, Bristol City Council
Dr Simon Bradley	Concord Medical Centre
Mr Tim Whittlestone	North Bristol NHS Trust
Geeta Iyer	BNSSG mass vaccination programme lead
Anne Morris	BNSSG mass vaccination programme lead

Supplementary file S2

Box S1: Examples of engagement and communication activities to enhance COVID-19 vaccine uptake among minority communities.

- 6 Short videos with community leaders from BAME communities sharing personal experience of how they have been affected by the pandemic and their worries about vaccination.
- Templated posters for fast turnaround production to be used locally – e.g. in clinics encouraging people to share their vaccination experiences; publicity for walk ins; which can include community leader quotes.
- Developing the Language Hub on the Healthier Together website including trusted resources in 22 different languages.
- Revised wording of COVID-19 Vaccine Appointment text message invitations from PCNs using more positively framed language and translated to 8 languages.
- Webinars developed for BAME and disabled communities, pregnancy, breast feeding & fertility, but accessible for all demographics.
- Small scale Webinars run by communities in own languages - such as in Somali community and by House of Praise, by local PCNs prior to the 1st dose clinics also helped to allay concerns and get people signing up for vaccinations once they became eligible.
- Videos and Leaflets in different languages
- Community led videos made by organisations embedded within local communities, often in community language.
- # vaccinated: Pull-up banners and posters encouraging people to share their experience of being vaccinated with family and friends were made available at all vaccination clinics
- Working with the voluntary sector, especially those which support asylum seekers and refugees, to share resources for vaccine clinic space, and community links to maximise clinic engagement

Box S2: Selected themes on COVID-19 vaccine perceptions from data collected through semi-structured interviews with homeless people in Bristol and Weston Super Mare in 2020

Prior to carrying out outreach activities with this population, BNSSG CCG conducted semi-structured interviews with 22 homeless people, in October- November 2020, in Bristol and Weston-Super-Mare, to explore their perceptions and attitudes towards COVID-19 vaccinations and how best to meet their needs.

The majority of those interviewed in Bristol reported that the coronavirus pandemic had little impact on them as their routines had stayed the same and they were still able to access food and similar support services. Two participants reported a positive impact of the pandemic on them as they had been offered shelter and accommodation.

Over two thirds of those that were interviewed were aware of a COVID-19 vaccine being developed. Over half of the people interviewed said that they would consider getting the vaccine if they were offered it, mainly because they believed having the vaccine will stop them getting the virus, will make them feel safe, and will allow them to see friends and family again.

"I will feel safer knowing I've had one and people will feel safer seeing me if they know I have had it. It means I might be able to see my friends and family again."

The majority of those interviewed would prefer for any information and advice to be communicated to them verbally, in a way that is simple and easy to understand by a trusted person. Trusted people included NHS health professionals such a doctor or nurse, as well as support workers and volunteers at local support services. Several people mentioned that having someone visit a support service site, to talk to them and answer any questions would be most beneficial.

"They have to prove it works. They need to convince me it works and it won't kill me. I want to see professionals, doctors or nurses, come and tell me about it and convince me. It cannot be written down as I can't read"

"Someone coming somewhere like here (Somewhere to Go) [43] to tell us information would be best. I have problems reading and understanding so would rather be told by someone what to do. I trust the Doctor and staff here for advice"

Box S3: Increasing the uptake of COVID-19 vaccine amongst homeless people in the BNSSG area.

Opportunistic, flexible approaches

- Combining vaccinating with surge testing
- Vaccinating off-JCVI (Joint Committee on Vaccination and Immunisation) cohort

Adapting to the population needs

- Taking the vaccine to homeless people – many will not go to a vaccine clinic. Staff needed to be out on the streets or going to their accommodation and working with them.
- Vaccination was a persistent, flexible offer for when it worked for them, with regular clinics available
- Incentives – food at vaccine outreach clinics and food vouchers given.

Using established networks and trusted people

- It is key to engage with staff in homeless/support settings to harness the potential of their strong relationships with homeless people
- Regular team meetings between the multiple partners delivering the outreach clinics to discuss progress and troubleshoot issues
- Good working relationships between commissioned and non-commissioned support services and commissioners

Working around practical issues

- Change in accommodation pattern from dormitory hostels to isolated (dispersed) accommodation. Outreach clinics may not be able to serve this group, so need to encourage registration and access through GP.
- Planning vaccine storage to avoid taking out individual vials
- Having access to Outcomes4Health (Pinnacle) to run reports on name and date of birth to check the person's vaccination status
- Considering issues with the NHS app in terms of communication for the second or booster doses; an NHS number or proving ID through photo of passport or drivers' licence may not be accessible for this group. People who were rough sleeping were provided with phones to ensure vaccine communication can reach them.

Box S4: Remaining challenges to identify and vaccinate homeless people in the BNSSG area.
Homelessness is often invisible in the healthcare or wider system

- The term "Homeless" is often used to define those rough sleeping rather than all those on the housing support register.
- Data about homelessness and where this is held is often an issue. Healthcare data held in NHS databases may not identify an individual as homeless due to poor coding practices. On the other hand, the housing support register and ABRITAS databases used by the local authority to identify people who are homeless may include, if at all, limited medical data.
- Many homeless people are not registered with GPs
- People may not have ID (issues for NHS app)
- People may have reasons for wanting to remain invisible e.g. No recourse to public funding, undocumented migrant

Multiple barriers in accessibility or acceptability of vaccination for this population

- Large number of people have experienced trauma and have an understandable mistrust in authority and statutory services
- Language barriers – especially if there is no recourse to public funding
- A population with complex needs which means engagement takes time and trust, and requires a persistent offer from services.

Ambiguous eligibility criteria for COVID-19 vaccination for homeless people and staff in support services

- Staff in the homeless sector can fall between cracks of social care and healthcare
- Eligibility for vaccinating homeless people was advocated on grounds of clinical vulnerability

Box S5: Increasing the uptake of COVID-19 vaccine among non-English-speaking people, people from minority ethnic groups, refugees, and asylum seekers:

Committed and passionate individuals are assets in their communities

- People from communities or with community links have worked tirelessly as advocators, action planners, influencers, vaccinators etc
- People have worked across organisational boundaries and collaborated for the cause, they have used years of experience and their established relationships and networks

Co-production with communities, and empowering them to lead on health promotion with NHS technical skills to support them

- Community leadership and action with community ownership: Mosque clinics were managing their own booking system on paper (to overcome wifi issues), with CCG support as needed for questions and concerns.
- Forums that flatten the hierarchy and communication chain between grass roots community groups and those with political power and influence.
- Using pre-existing networks of communities and professionals, and having community members within the vaccination programme
- Talking to people in the community to learn what the issues were, including investing time at clinics and engagement events through the Insight team.

Communicating honestly, bilaterally and in relevant ways

- People delivering the messages who had constituency with that community: e.g. religious leaders doing webinars
- Using channels that are relevant to this population e.g. BCFM radio, DUNYA, videos on Healthier Together website, Somali Facebook groups, and others chosen by the community
- Sharing programme updates with communities as partners
- Being accessible e.g. phone number available for conversations about vaccine concerns
- Adapting the messaging style away from traditional healthcare messaging, e.g. healthcare professionals talking about their personal fears around the vaccine.

Digital ways of working

- Meetings on teams and zoom to be accessible to people who couldn't travel or were based in communities
- Webinars: what is fact, what is fiction and what is opinion? The recordings were shared through twitter, and community networks.
- WhatsApp conversation groups for community leaders to network
- Encouraging people to share their vaccination experience e.g. vaccine selfies on social media

Fitting the approach to the community needs

- Flexible appointments so people can come for the vaccine when they can/need
- Understanding that information and influence flows through networks of family and friends, as well as through community leaders
- Outreach: taking healthcare to the community and vaccinating out of JCVI cohort at outreach clinics
- People with trusted relationships with communities doing outreach: trusted community workers going to clinics to vaccinate, and familiar GPs present at outreach clinics.

Box S6: Barriers to working effectively with minority ethnic communities to increase vaccination uptake:

Historical and institutional barriers to ethnic minority groups' engagement with healthcare and local authority organisations

- Acknowledging the root causes of low vaccine uptake: There are strong sentiments of mistrust in the system for some individuals and communities. Although some have been empowered by the work, others are suspicious of the sudden interest in their community
- Reviewing how well BNSSG partners serve minority populations and how do they integrate into communities
- Consider the extent of diversity in the BNSSG workforce, and in senior healthcare leadership positions, to ensure meaningful engagement with communities.

Communications: What you say and how you say it matters.

- Some media and organisational key message "we're not quite sure why" about the low uptake in certain groups is unhelpful. It avoids acknowledgement of historical and current issues faced by these groups, and misses opportunities to highlight the root causes of low engagement and uptake.
- Webinars suit some people, but may not suit others (who may be digitally excluded), and/ or the webinars are facilitated by healthcare professionals who haven't earned trust from the community.
- Early posters and communications did not represent ethnic minorities well, so people did not engage with them. Furthermore, many do not read the language they speak. Videos in multiple languages can be useful to overcome this barrier.
- Social media can be a powerful source of misinformation, and hence, should be used to counteract this content using materials with the right content and in the right style.
- When communicating with communities, BNSSG partners need to ensure that they are not talking to communities, but rather, talking with them.

Practical barriers

- Limited access to the single vaccination bus for flexible mobile clinics
- Digital poverty in communities
- IT and staffing issues at smaller vaccination centres

One size does not fit all; the process of engagement needs to be tailored to minority groups

- Little success of engagement activities with the Eastern European communities: Church faith leaders were less ready to engage in general. There was widespread anti-vax messaging in the communities. The strategy to engage with shops to set up clinics was not successful since those were actually owned by members of community. It may be more effective to map out workplaces and attempt to approach those communities for engagement in the workplace.

Box S7: Increasing the uptake of COVID-19 vaccine among Gypsy, Roma, Travelers (GRT) and Boatpeople

- The design and delivery of outreach clinics and engagement materials required the involvement of key healthcare professionals with a good understanding of communities of interest that are not geographically co-located. These included the expertise of midwifes and nurses who have had previous relationships with those communities and understand the differences between these communities
- Taking the vaccine promptly to GRT sites at times that work for them. Small teams of vaccinators kept attending these sites on a weekly or fortnightly basis, continuing to offer vaccination.
- Recognising elders (especially matriarchs) as influencers in their communities and families and engaging with them to inform communication approaches
- Including council GRT liaison workers in outreach activities to provide marshalling, practical support, and signposting people to vaccinators
- Tailoring communication to the population, incorporating literacy limits and using their language. For example, many in these communities prefer to use the term "needles" instead of "vaccines".
- Understanding and respecting that some members of these communities do not want to be on the radar. Therefore, it was important to allow them to stay "off the radar"; it was important to clearly explain to them that they did not need to register with a GP to get vaccinated.
- The outreach clinics delivering COVID-19 vaccination to boating travelers had to move across geographical boundaries e.g. into Bath, Swindon and Wiltshire area, to accommodate the regular travelling of this community. A boat delivering vaccination service ensured that the needs of this community were met.

Supplementary file S3

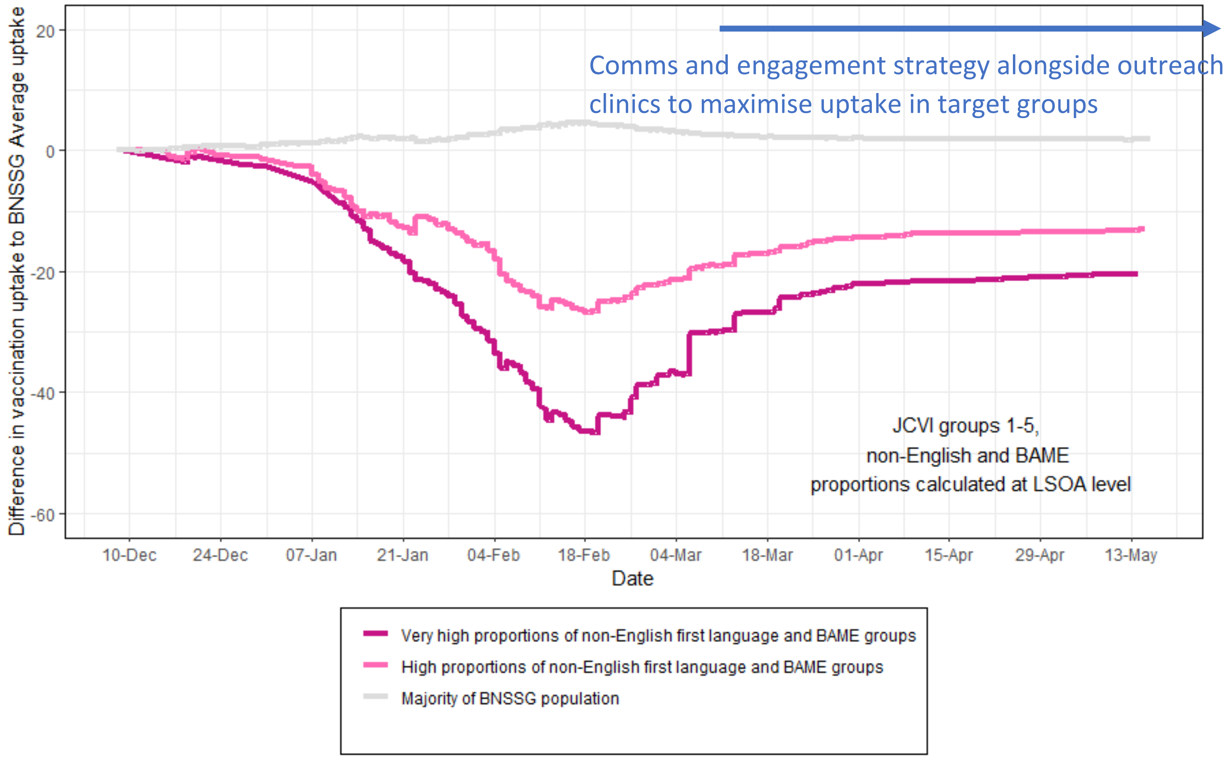

Figure S1. Uptake of COVID-19 vaccination for small areas (LSOAs) of BNSSG with higher proportions of people with non-English first language or from ethnic minority groups (referred to as Black Asian Minority Ethnic in this figure), for JCVI (Joint Committee on Vaccination and Immunisation) cohorts 1-5.

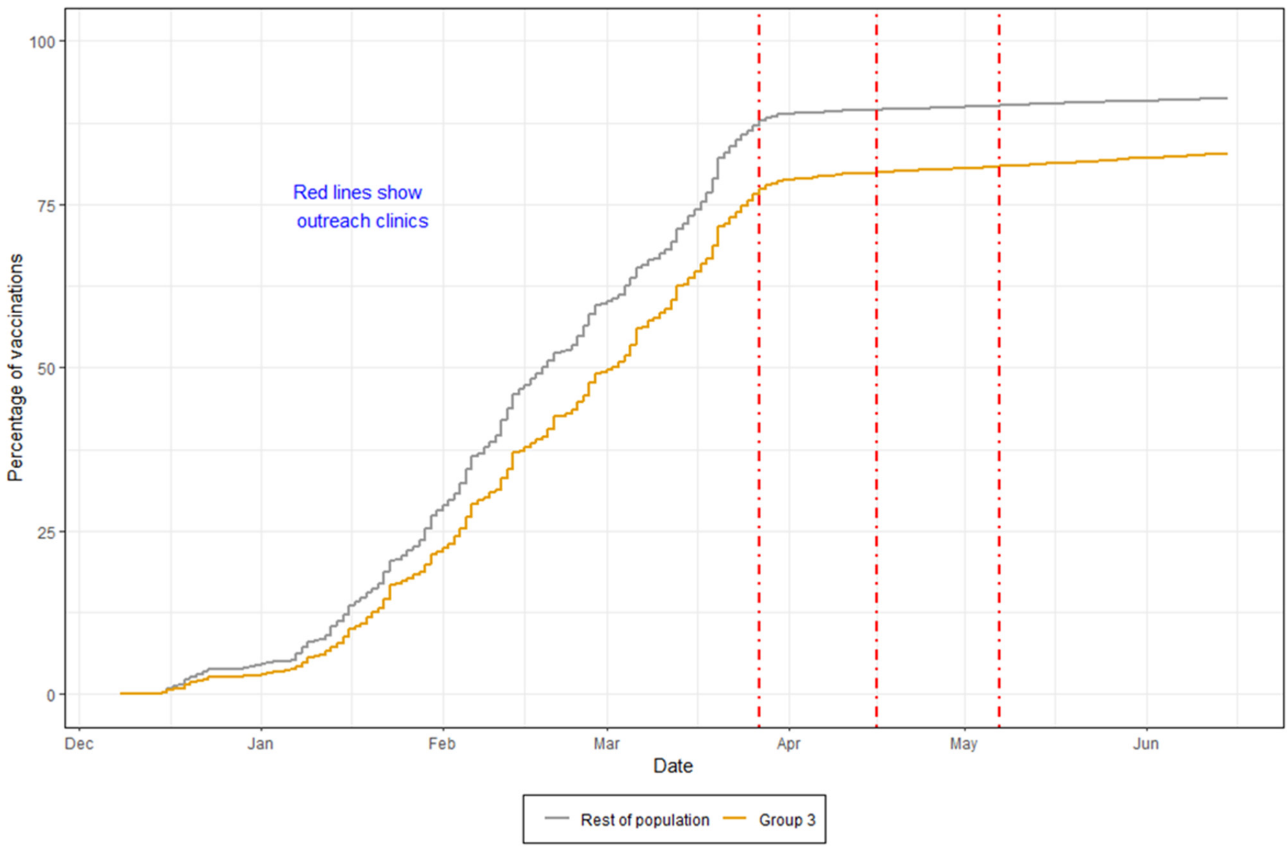

Figure S2. Percentage vaccination uptake (1st dose) among Group 3 in BNSSG area. The red dotted lines represent some of the outreach activities. Group 3 includes GRT population, those living a distance from the vaccine centre (road distance to a GP surgery indicator >= 5.5km in the system wide dataset), those living in areas of high deprivation (IMD quintile of patient LSOA = 1) and rural communities. Around 76.8% of the population in this group had received first doses (to 17th May 2021, cohorts 1 to 9), compared to 84.4% of the BNSSG population. *IMD: Index of Multiple Deprivation LSOA: Lower layer Super Output Area*
